# GC and Repeats Profiling along Chromosomes—The Future of Fish Compositional Cytogenomics

**DOI:** 10.3390/genes12010050

**Published:** 2020-12-31

**Authors:** Dominik Matoulek, Veronika Borůvková, Konrad Ocalewicz, Radka Symonová

**Affiliations:** 1 Faculty of Science, University of Hradec Kralove, 500 03 Hradec Králové, Czech Republic; dominik.matoulek@uhk.cz (D.M.); veronika.boruvkova@uhk.cz (V.B.); 2 Department of Marine Biology and Ecology, Institute of Oceanography, Faculty of Oceanography and Geography, University of Gdansk, 80-309 Gdansk, Poland; konrad.ocalewicz@ug.edu.pl; 3 Department of Bioinformatics, Wissenschaftszentrum Weihenstephan, Technische Universität München, 80333 Freising, Germany

**Keywords:** AT/GC heterogeneity, chromosome banding, fish cytogenetics, GC-profile, repeats organization

## Abstract

The study of fish cytogenetics has been impeded by the inability to produce G-bands that could assign chromosomes to their homologous pairs. Thus, the majority of karyotypes published have been estimated based on morphological similarities of chromosomes. The reason why chromosome G-banding does not work in fish remains elusive. However, the recent increase in the number of fish genomes assembled to the chromosome level provides a way to analyse this issue. We have developed a Python tool to visualize and quantify GC percentage (GC%) of both repeats and unique DNA along chromosomes using a non-overlapping sliding window approach. Our tool profiles GC% and simultaneously plots the proportion of repeats (rep%) in a color scale (or vice versa). Hence, it is possible to assess the contribution of repeats to the total GC%. The main differences are the GC% of repeats homogenizing the overall GC% along fish chromosomes and a greater range of GC% scattered along fish chromosomes. This may explain the inability to produce G-banding in fish. We also show an occasional banding pattern along the chromosomes in some fish that probably cannot be detected with traditional qualitative cytogenetic methods.

## 1. Introduction

Classical chromosome banding methods such as G- (Giemsa), R- (reverse) and Q- (quinacrine) banding allow for routine chromosome analysis in higher vertebrates, including human clinical cytogenetics [1,2,3], and many more. A recent review of these heterogeneous chromosomal bands and sequence features is available [4]. A fully different and incomparable situation exists in lower vertebrates, particularly in fishes. Compared with other vertebrates, fish have smaller chromosomes and a narrower range of GC% values in entire genomes [5,6]. Despite numerous attempts, e.g., [7,8,9], the chromosome banding methods mentioned above do not yield usable patterns in fish. The research performed up to now was summarized concluding that C-banding [10] and silver-staining [11] in fishes provide reasonably good results, whereas very little success has been achieved using G-bands [12]. The only way to produce a reliable pattern on fish chromosomes was the application of replication labelling, as different regions of genome replicate at different moments during S phase of the cell cycle [13]. The replication banding utilizes the incorporation of a thymidine analogue, 5-bromo-2′-deoxiuridine (BrdU), into nuclear DNA during the S-phase of DNA replication. Then regions with BrdU are visualized by detection on metaphase chromosomes. Bands with incorporated BrdU may be revealed, for example, by Hoechst 33,258 fluorescence, acridine orange fluorescence, or fluorochrome-photolysis-Giemsa staining (FPG), among others [14]. It has been shown that heterochromatic, AT-rich G-bands and C-bands are late replicating, while euchromatic, GC-rich R-bands replicate early during the S-phase [2]. Despite its high resolution in mammals, replication banding patterns have been produced in a limited number of fish species so far. Application of FPG enabled the identification of early and late replicating chromosomal regions with high resolution banding patterns in salmonids [15,16], white sturgeon [16], and eels [17,18]. Less clear patterns after BrdU incorporation were observed on chromosomes of cyprinids [19,20], anastomids [21], ictalurids [22], flatfish [23], pufferfish [24] and characids [25]. The interspecies differences in the resolution of the replication banding may result from different genome composition and the size of chromosomes. Salmonid genomes are of polyploid origin and have relatively large chromosomes that are favourable for the distinct and clear replication banding pattern [16]. However, even this laborious procedure applied in fish did not always produce results comparable with those in mammalian and avian cytogenetics [12]. The presence of very small microchromosomes along with larger macrochromosomes in some basal fish lineages (chondrichthyans, sturgeons, gars) resembling those in birds and some reptiles complicate fish cytogenetics even more because of their indistinguishable chromosome morphology.

Values of GC% are associated with numerous traits including gene density, chromatin structure, the proportion and types of transposable elements, DNA replication timing, nucleosome formation potential etc. [26]. To test whether GC content differences might explain the lack of G-bands in fish, we investigated the fine-scale AT/GC organization in fish. Thanks to the increasing availability of fish genomes assembled to the chromosome level and at the same time of their soft-masking, i.e., labelling repetitive elements as the lower case in the otherwise upper case represented DNA sequence, it is possible to produce a virtual banding pattern of GC% and repeats percentage (rep%) along chromosomes. The recently published genomes of sterlet sturgeon [27] and reedfish [28] were important milestones for fish compositional cytogenomics sensu [29] together with the immense body of evidence accumulated by traditional cytogenetics. In the traditional (i.e., qualitative) fish cytogenetics, there are two mutually non-exclusive ways to visualize GC% and rep% even on the same metaphase. These are the CDD-staining combining AT- and GC-specific fluorochromes to the same metaphase [30,31] for GC% and fluorescence in situ hybridization (FISH) with a repetitive DNA fraction, e.g., cot-1, as a probe [32] or a more destructive visualization of constitutive heterochromatin using C-banding [10,33] for rep%. However, application of these methods is limited in fish due to the small size of their chromosomes. Moreover, it requires time-consuming laboratory processing including chromosome preparation from living fish. On the other hand, cytogenetic methods including C-banding and DAPI-staining usually enable identification of the centromeres, which is not yet possible in most of fish genomes.

To the best of our knowledge, there is no such specialized bioinformatics tool available to integrate and plot both GC% and rep% into a single image. There are some tools producing GC-profiles along chromosomes, e.g., [34,35], or tools integrated, e.g., in Bioconductor plotting diversified features along chromosomes [36] but never plotting simultaneously the proportions of repetitive DNA together with the GC% of non-soft-masked (non-repetitive) and soft-masked (repetitive) DNA.

Our aims were: (1) to assess differences in compositional organization (GC and repeats proportions) of chromosomes at multiple levels of resolution (i.e., with different sliding window sizes) among vertebrates with a focus on fishes; (2) to utilize the increasingly available genomic data on the chromosome level and their constantly increasing quality; (3) to virtualize the traditional qualitative molecular cytogenetic methods in silico; (4) to assess the role of transposons and other repetitive elements on the entire AT/GC composition along chromosomes; and (5) to produce a publicly available tool visualizing and quantifying these two major features (GC and repeats proportions) along chromosomes assembled to the chromosome level.

Producing two types of plots, combining a color scale with percentage values along chromosomes with a customized non-overlapping sliding window size helped to resolve the conundrum of unavailability of banding patterns in fish cytogenetics. Namely, the fine-scale organization of repeats and their own GC content homogenize the overall GC% along fish chromosomes, preventing the formation of larger regions with an elevated GC% separated by sharp borders.

## 2. Materials and Methods

### 2.1. Data Acquisition and Processing

Altogether, we utilized genome assemblies of 41 fish and one tunicate species (Table A1) assembled to the chromosome level available in the database Ensembl (37 species with already available soft-masking; Release 100; [37]) and in NCBI six species which genomes had to be processed with soft-masking software [28], e.g., using the online tool RepeatMasker version 4 [38]). These species include one tunicate (*Ciona intestinalis*), three chondrichthyan species, three non-teleost ray-finned fish, i.e., one reedfish (*Erpetoichthys calabaricus*), one sturgeon (*Acipenser ruthenus*) and one gar (*Lepisosteus oculatus*), and 35 teleosts. To compare fish GC% and repeats organization along chromosomes with mammals, we further utilized genome assemblies of gorilla, cat, little brown bat, and greater horseshoe bat, also available already soft-masked in Ensembl. We compared three different non-overlapping sliding window sizes with 1 kbp as default. Furthermore, we tested non-overlapping sliding window sizes 3 kbp and 10 kbp in selected species. This is highly relevant for polyploid (e.g., salmonids) or (extremely) large (reedfish, zebrafish) fish genomes. The sliding window size 3 kbp reflects the fact that mammalian genomes are about three times larger than fish genomes, while both converge on approximately 2n = 46–50. This enabled us to compare fish and mammalian chromosomes at a corresponding scale.

### 2.2. DNA Profiling Tool

The tool called EVANGELIST (=EVAluatioN on GEnome LIST) utilizes the non-overlapping sliding window (referred to as sliding window below) approach to quantify and visualize the percentage of repeats and GC percentage (GC%) in both repeats and non-repetitive DNA simultaneously. It includes the following Python components: DNA_puller, gnuplot_generator and a set of Jupyter notebooks. To run this tool, it is necessary to have the BioPython [39] library installed. The tool performs four basic steps to produce the presented results:

1. Data download from a database such as Ensembl or NCBI, where they are accessible by the FTP. The tool saves data for every requested species into its own folder and unzips them.
2. Data analysis by the sliding window approach is performed for each FASTA file separately with “DNA_puller”, a component provided on GitHub. Each window position yields the number of occurrences of each letter (i.e., ATGC), discerning the upper and the lowercase ones.
3. The raw data are processed as a preparation for charts, giving GC% and the ratio between soft-masked (identified repeats) and non-soft-masked (non-repetitive DNA or not identified repeats) DNA in a CSV file for each chromosome. Such a file has three columns (index, i.e., position in DNA, GC%, and ratio) and a generally high number of rows, each of which will present a point in the chart. For instance, for a chromosome with 10 Mbp and a sliding window of size 1kbp, the result file has 10 Mbp/1 kbp = 1000 rows hence 1000 points in the chart.
4. Generation of the definition files and rendering charts is a two-step process performed with the tool GNUplot, version 5.2. The former is executed with our component “gnuplot_generator”. During this step, the CSV files are sorted by the number of lines counted by the wc (‘word count’) program in Linux. Finally, the charts are rendered.

### 2.3. Plotting Large-Scale Profiles and Statistical Analyses

Plotting extremely large chromosomes presented a crucial issue. The size of “normal” (macro)chromosomes ranges from 15 to 150 Mb. To prevent information loss, our tool produces plots with a tailored size according to chromosome sizes in each species separately. This ensures that each set of chromosomes is plotted as large as possible, which is crucial because of the requirements to visualize an extreme number of points: e.g., the largest chromosome in Northern pike (average C-value 1.1 pg [40], and average assembly size 921 Mbp; GenBank) is the linkage group (LG) 11 with size 55.41 Mbp, meaning that 55,410 points have to be visualized for this single chromosome (each point represents 1000 bp or 1 kbp). The complete set of chromosomes in this species is 10,000 × 25,000 pixels large and the file size is about 10 MB. On the other hand, the scale differs in each species.

We have tested the obtained results for GC% and repeats% for the linear relationship and correlation between these two measures in all species under study using BioPython [39].

Icons made by https://www.flaticon.com/authors/freepik.

The tool is available on GitHub https://github.com/bioinfohk/evangelist and the complete collection of all profiles produced in the framework of this study and in full resolution is available on the link https://github.com/bioinfohk/evangelist_plots.

## 3. Results

**Figure 1:**
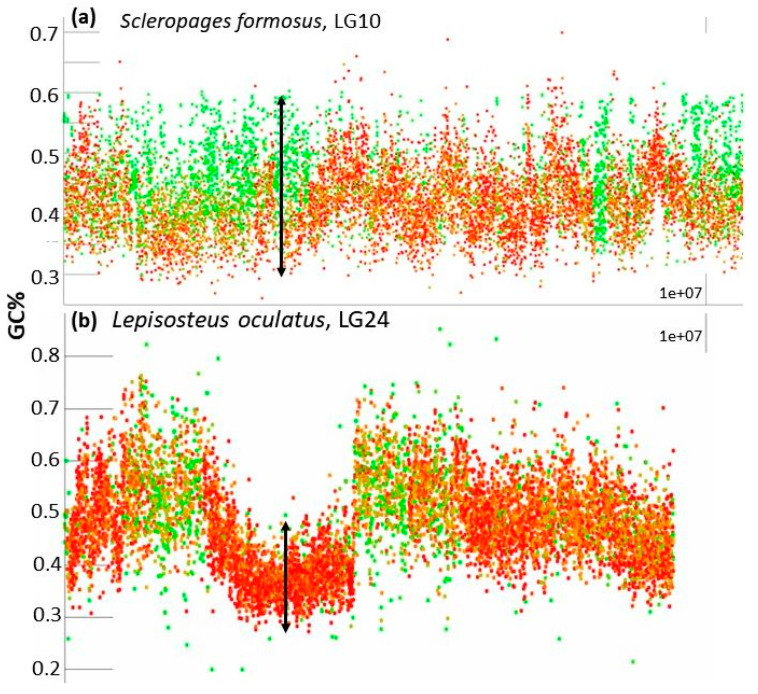
Details of parts of chromosomes of two representative fish species produced with the default setting of the non-overlapping sliding window size 1 kbp. (**a**) Asian arowana (*Scleropages formosus*), where the soft-masked DNA, i.e., repeats (green) attain high GC% whereby they homogenize the overall GC content to form a flattened upper bound of GC%. Here, the wide range of GC% values in repeats is also apparent and shows the importance of the small window size used here as default; (**b**) a different situation exists in the spotted gar (*Lepisosteus oculatus*) with one GC-poorer non-soft-masked (i.e., non-repetitive DNA, red) region surrounded by regions with a sharply elevated GC%. Each dot represents a single sliding window value of GC% (y axis) and soft-masked (repetitive) DNA percentage (red no repeats, green 100% repetitive, orange approx. 50% of repetitive DNA). The arrows in both images indicate a greater range of values of GC% in Asian arowana (**a**) than in the selected region of the spotted gar (**b**).

**Figure 2:**
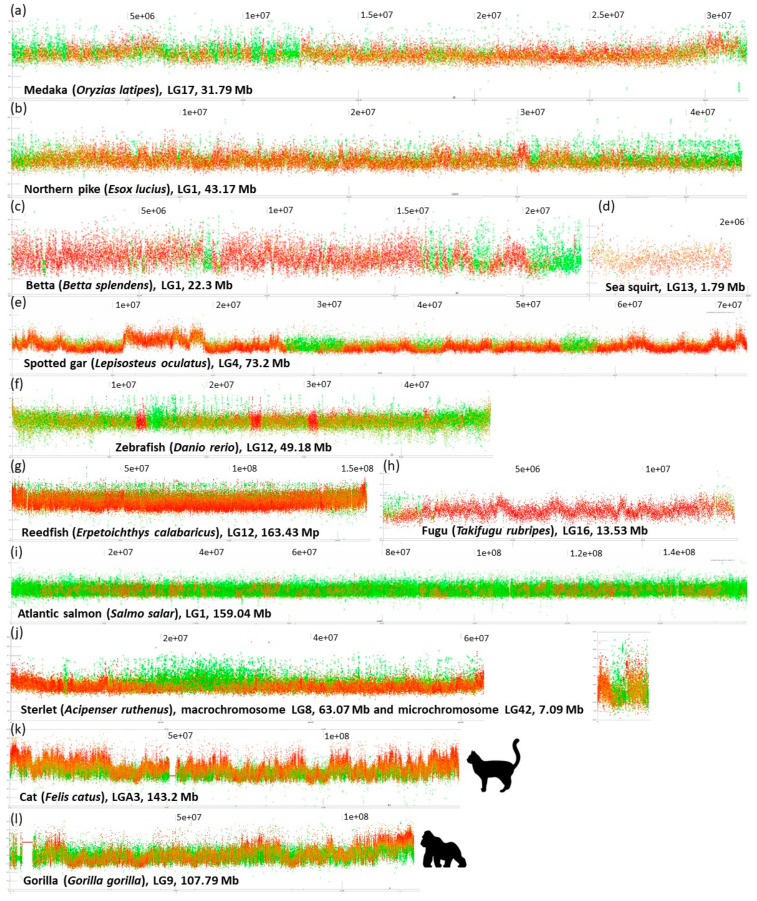
Graphs of mostly middle-sized chromosomes (unless otherwise indicated) with the default setting of the sliding window size 1 kbp. (**a**) Medaka shows repeats intermingled with unique sequences resulting in an overall orange coloration alternating with prevailing repeats (green) and unique (red) regions; (**b**) northern pike, with all acrocentric chromosomes, the largest chromosome shown; (**c**) betta with repeats localized in interstitial blocks and at a single end of the chromosome resulting in an overall red coloration; (**d**) sea squirt with homogeneous GC-poor DNA, the smallest chromosome shown; (**e**) spotted gar, the only fish so far known with the AT/GC heterogeneity, the largest chromosomes shown; (**f**) zebrafish, an example of an extremely GC-depleted fish genome with almost no fluctuations; (**g**) Reedfish with extremely large chromosomes without any prominent fluctuations in GC%; (**h**) fugu, a short linkage group (LG) with an extremely reduced amount of repeats; (**i**) Salmon, a polyploid AT-rich genome, the largest chromosome shown; (**j**) Sterlet, another polyploid fish with AT-rich(er) macro- and GC-rich(er) microchromosomes; (**k**) cat and gorilla (**l**) are mammalian outgroups with GC- and gene-rich peaks and rather AT-rich repeats. Complete plots of all analysed species are available at our online repository https://github.com/bioinfohk/evangelist.

**Figure 3:**
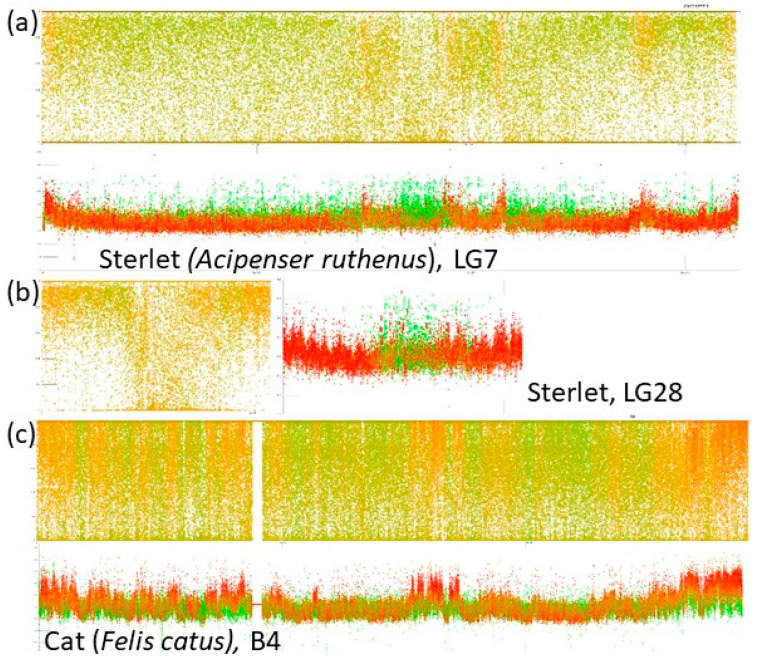
Comparison of the two major options of setting of GC% and rep% visualization with our tool with the default sliding window size 1 kbp. (**a**) One macrochromosome of sterlet, where GC% is represented by the color-scale mimicking the CMA_3_-fluorescence staining in the upper panel (GC-rich in red, AT-rich in green) compared with the swapped setting, where GC% is plotted as a profile and rep% as the color-scale in the lower panel; (**b**) the same for one microchromosome of sterlet; (**c**) B4 of cat as an example of a mammalian LG.

In the default setting, our tool plots GC% along chromosomes as points representing each consecutive 1000 bp (1 kbp) with 0–100% of GC on the y axis (Figure 1). The percentage of repeats (rep%) is plotted as a color gradient of these points, where green represents 1 kbp of soft-masked DNA, i.e., 100% of repeats, and red represents 1 kbp of non-soft-masked DNA, i.e., no repeats detected within the range of these 1 kbp (Figure 2). Our efforts to produce graphs as informative as possible resulted in very large plots. We have chosen this setting as the primary one because of its higher information value. This pattern of GC% values and colors can be easily swapped so that the scale of GC% can actually mimic the CDD-staining on chromosomes, where GC-rich regions are red and AT-rich regions are green and the rep% is on the y axis (Figure 3).

### 3.1. GC-Profiles in Fish

Regarding the GC% values, the sliding window size 1 kbp proved to yield the best resolution and the fish species analysed so far produced the following patterns:

1. The entire chromosome is formed by a generally flattened range of points with GC% between the minimal values around 35% and the maximal values around 55% (*Oryzias latipes*, Figure 2) or sometimes 30–60% (*Betta splendens*, Figure 2) with only rare or occasional slight departures from this pattern. Whereas some species show a narrower GC% range with almost no fluctuations/departures, e.g., in the Blunt-snouted Clingfish (*Gouania willdenowi*), some other species show an even broader range of GC% 30–65% with some more prominent local elevations or depletions of GC% (*Scleropages formosus*). Occasional slight elevations in GC% occur at the ends of chromosomes.
2. No prominent pattern occurs in the basal chordate (tunicate) sea squirt (*Ciona intestinalis*). This pattern can be ascribed to an extremely low amount of DNA in the chromosomes (4.5–10 Mb). The majority of points occur in the range 30–40% of GC with only very rare and narrow peaks or isolated points reaching 50% of GC.
3. So far, the only known fish species with heterogeneous AT/GC organization along LGs is the spotted gar (*Lepisosteus oculatus*, Figure 2). Here, a rather narrow “baseline” of densely organized points of GC% between 30–50% alters with sharp and compact peaks reaching over 60% of GC%.
4. Another extreme situation exists in the reedfish (*Erpetoichthys calabaricus*, Figure 2) with a dense organization, however, resulting in a flat range of values between 30–55% GC. This flattened appearance can be ascribed to the exceptionally large size of chromosomes (88.37–350.1 Mb) that are even larger than mammalian chromosomes (gorilla 32.72–219.76 Mb).
5. More fluctuating GC% values exist in tetraodontid fish with reduced genome size (*Tetraodon nigroviridis*, *Takifugu rubripes*, Figure 2; [41,42,43]) and to some extent in other species with reduced genomes e.g., the three-spined stickleback (*Gasterosteus aculeatus*).
6. A combination of a flattened range of GC% values in large(r) chromosomes (i.e., macrochromosomes) and more or less clear GC% elevations in smaller chromosomes (i.e., microchromosomes) exists in the sterlet (*Acipenser ruthenus*, Figure 2j) and all three chondrichthyan species analysed (*Amblyraja radiata*, *Chiloscyllium plagiosum*, and *Pristis pectinata*). Here, with the decreasing chromosome size, elevations in GC% firstly appear at the ends of chromosomes. In smaller chromosomes, internal GC% fluctuations occur.

### 3.2. Repeats Content and Organization in Fish

The default sliding window size of 1 kbp proved to yield the best resolution relative to repeat distribution along chromosomes. The following patterns and their mutual combinations have so far been observed:

1. Blocks of repeats prevailing over the non-repetitive DNA at both ends of chromosomes. This pattern is particularly prominent in species with all acrocentric chromosomes (e.g., *Esox lucius* (Figure 2; [44]), *Oreochromis niloticus* [45], *Sparus aurata*, etc.). The size of these blocks of repeats varies within and among species.
2. Interstitial, clearly delineated small blocks of almost exclusively repetitive DNA. (e.g., *Betta splendens*, Figure 2, *Ictalurus punctatus*, *Scleropages formosus*, *Oryzias latipes*).
3. Dispersed and intermingled repeats occurring mostly in fish species with larg(er) genomes (e.g., *Danio rerio*, *Astyanax mexicanus*, and pseudotetraploid salmonids *Oncorhynchus mykiss* and *Salmo salar*). Here, either completely green or orange regions of varying size are interrupted with small blocks of non-repetitive DNA.
4. Limited extent of repeats proportion caused by reduced genome size through repeats elimination (*Tetraodon nigroviridis*, *Takifugu rubripes,* Figure 2*, Gasterosteus aculeatus*) or through insufficient repeat-masking (*Oryzias javanicus*, *Scophthalmus maximus*, etc.).

These patterns of repeats distribution can combine and co-occur in a single fish species. However, it is necessary to stress that these patterns depend on the quality of soft-masking that is linked to the genome assembly quality. Hence, the obtained patterns cannot be considered ultimate in genomes, where soft-masking revealed only a smaller fraction of repeats.

Interestingly, in regions, where GC% decreases in the non-repetitive fractions, the GC% of repeats increases and thus compensates for this decrease, keeping the overall GC% values with a flattened upper bound, e.g., in Figure 1b in Asian arowana, Figure 2a medaka, Figure 2b the Northern pike or Figure 2c betta. More fish species showing this phenomenon can be seen on our GitHub repository. In regions, where non-repetitive DNA becomes fully absent, the repetitive DNA follows the GC% of the non-repetitive fraction from the surrounding regions. This prevents the formation of peaks with a higher GC% and of sharper borders in GC%.

The inverted representation of GC% and rep% shown in Figure 3 was produced to enable a direct comparison with cytogenetic CMA_3_ staining. This helps to understand why this AT/GC-based CMA_3_ staining does not work in fish–the GC-rich regions are too small and less prominent to be recognizable on small fish chromosomes.

### 3.3. GC- and Repeat-Content in Selected Mammals and Comparison with Fish

A fully different picture exists in the four representatives of mammals (gorilla, cat, little brown bat, and greater horseshoe bat). Here, the flat “baseline” is formed by a mixture of repeats and non-repetitive DNA (orange points), whereas the highly GC-enriched genomic fractions are formed by clearly gene-rich DNA and the GC-depleted fractions mostly by repeats. The gene- and GC-rich regions form sharp borders and clearly delineated peaks along the chromosomes. There are some repeats with a higher GC%, however they hardly reach the GC% of gene-rich DNA and never form peaks as the gene-rich DNA does. Hence, there are no regions of GC-rich(er) repeats as described above in fish.

### 3.4. Different Sliding Window Sizes in Fish and Mammals

**Figure 4:**
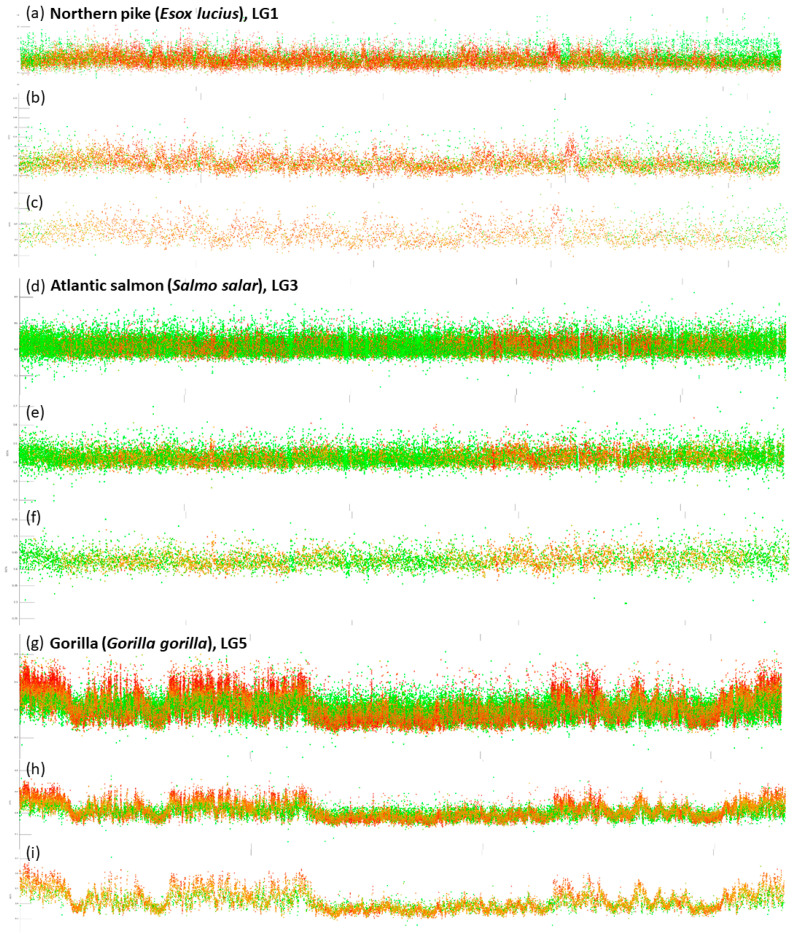
Comparison of three consecutive sliding window sizes, i.e., 1, 3 and 10 kbp in vertebrates with substantially different genome size. The Northern pike (**a**–**c**); the Atlantic salmon (**d**–**f**); gorilla (**g**–**i**).

Since fish genomes are mostly up to three-times smaller than the mammalian ones but both groups converge on approximately 2n = 46–50 chromosomes, mammalian chromosomes are larger. Similarly, genomes of polyploid fish are substantially larger. This is reflected in our tool by the possibility to select one of three currently available sliding window sizes (1 kbp, 3 kbp, and 10 kbp). Examples of results with these three different sliding window sizes are shown in the Figure 4. Following species are compared: one fish with a typical teleost haploid genome size around 1 pg, the Northern pike, one polyploid fish with the genome size around 3 pg, the Atlantic salmon and one mammal with genome size 3.5–4 pg, the gorilla. The sliding window size 1 kbp appears the best suitable for teleosts and other species with a comparable genome size. The sliding window size 3 kbp appears suitable for polyploid fish and mammals and better enables downsizing of resulting plots. The sliding window size 10 kbp can be used in the best way when an extreme downsizing of the plots is required or in species with (extremely) large genomes (e.g., amphibians, reedfish, mammals or other organisms including highly polyploid plants).

### 3.5. Relationship between GC% and Repeats Percentage in Fishes and Mammals

Our tool enables a fast extraction of the values of GC% and rep% for each sliding window analysed (represented as a dot in the plots), makes scatterplots of these two measures and calculates Pearson’s correlation coefficient (r). Separately, we tested for the linear relationship and correlation between these two measures in all species under study. This analysis shows a weak but significant positive correlation (r = 0.1–0.225, *p* = 10^−16^) between GC% and rep% in nineteen of the 42 fish or fish-like species with the exception of *Amphiprion percula*, where r = −0.172. In the remaining fish species, r < 0.1 and in eight of them r = −0.082–−0.029, *p* = 10^−16^–10^−6^). These nineteen fish species show now phylogenetic relatedness. In the four mammals tested, there was a weak but significant negative correlation (r = −0.226–−0.046, *p* = 10^−16^) between GC% and rep%. Data quality (either soft-masking or genome assembly) was insufficient for the following four species (*C. plagiosum*, *A. radiata*, *G. morhua*, *P. pectinata*). It is necessary to say that this analysis is highly dependent on the repeat masking quality and its accuracy will be increasing in the future.

Scatterplots including the r values for each species are available at our GitHub repository https://github.com/bioinfohk/evangelist_plots/tree/master/rep%25_vs_GC%25.

### 3.6. Functionality of the Tool

What makes this tool useful is the fully automated approach to data analysis. All steps are performed by a computer without any need of user´s input. The user only provides the names of species and waits for some time that depends on the bandwidth and the provided computer.

## 4. Discussion

### 4.1. Technical Requirements and Limitations

The presented plots shown here were created using a Linux server (64 GB RAM) however, the tool can run on a standard desktop computer only with a longer waiting time. The tool is fully dependent on the quality of the input data. This is the genome assembly quality and the quality of the repeat-(soft)masking (RM) procedure. RM can be redone in older genome assemblies against any up-to-date and/or custom repeat libraries in a separate step using, e.g., RepeatMasker tool [38]. We assume that the newly available genome assemblies will have increasingly better RM quality because of the rapid development in the masking strategies and the number of repeats newly identified. Currently, it is always necessary to bear in mind what might be the available level of RM of each species and hence until what extent the RM was sufficient, e.g., the very low rep% in *Tetraodon nigroviridis* might be indeed ascribed to its extremely streamlined genome with eliminated TEs [43]. Similarly, the high rep% in salmonids or zebrafish can be ascribed to their large genomes full of TEs [42,43]. On the other hand, the rep% in *Oryzias javanicus* is far more reduced in comparison with its much more explored congener *O. latipes* (Figure 1a) or another well explored model species *A. mexicanus* [43]. This means that the genome assembly and/or RM quality in *O. javanicus* is substantially lower than in other species.

There are several types of resulting plots based on the resolution of these considerable datasets: (1) A3-format; (2) large-scale plots; (3) crops; and (4) a combination of the previous ones.

Linking of chromosomes with their corresponding linkage groups (LGs) from genome assemblies is available only for a few fish species and this appears to be another limitation of LG profiling in practice. This means that in fish, it is mostly impossible to deduce the chromosome morphology (meta- vs. acrocentric, etc.) from the GC and repeats profiles at this stage. So far, we depend on the comparison of size-sorted LGs with the subjective size of chromosomes from cytogenetic studies and/or on the usage of genome browsers (e.g., the recently released NCBI Genome Data Viewer) to identify potential centromeres along LGs. This means that we can only estimate the position of centromeres after the comparison with chromosome size and morphology. How we could proceed with the identification of centromeres further depends on the quality of genome assemblies that is however increasingly better, particularly thanks to long-read sequencing and its combination with the more accurate short-read sequencing (the hybrid approach). Another possibility is to localize genes for nuclear ribosomal RNA in the genome browser and on chromosomes.

### 4.2. GC- and Repeats-Profiling and Chromosome Banding in Fish

Replication banding has been used in fish to assign chromosomes to their homologous pairs [46], to identify sex chromosomes [21,47], and to describe chromosome rearrangements and polymorphisms [15,46]. It worked well on large salmonid chromosomes [16,46], but it is less applicable to small cyprinid or poecilid chromosomes [48]. On the other hand, the application of replication banding may be limited not only by the chromosome size, the degree of their spiralization but also by the genomic composition. A distinct and quite clear replication banding pattern has been observed in salmonids, whose repetitive DNA accounts for up to 60% of the genome [49]. On the contrary, a reduced number of replication bands was recognized along pufferfish chromosomes [24], whose genomes contain less than 10% repetitive elements due to their compaction [50,51]. Comparison of the replication banding pattern on the chromosomes of rainbow trout or masu salmon [16] and pufferfish clearly shows that salmonid chromosomes exhibit many early and late replicating bands alternating along their chromosomes [16], while pufferfish chromosomes are mostly composed of large early replicating bands sometimes covering almost entire chromosomal arms and small late replicating bands restricted to centromeric regions [24]. Genomes of salmonids and pufferfish underwent different (opposite) evolution, namely, whole genome duplication and genome compaction, respectively, that affected AT/GC composition in these fishes. This can be clearly observed in the GC-profiles of LGs studied in these species in the present research (Figure 2). In rainbow trout and salmon, repetitive DNA is equally distributed in the genome and interrupted with small blocks of non-repetitive DNAs while, in pufferfish most of the genome is composed of non-repetitive DNAs (Figure 2) given that repeat masking was of comparable quality in these species. This shows that the reduction of repetitive genomic elements during evolution decreases the resolution (and efficiency) of chromosomal banding based on the different phases of replication. GC% and repetitive DNAs profiling described here may indeed become an efficient tool in approaching “computational cytogenetics” in the future because this compensates for the small sizes of teleost chromosomes. Hence this approach might be complementary to the replication banding in species with suitable genomes/chromosomes.

Our results are consistent with previous findings that the GC% of the repetitive (soft-masked) genomic fraction is mostly higher than the genome-wide GC% in fish [52]. Namely, our plots in fish show that the repetitive fraction homogenizes GC% (compensates for the decrease in GC% of the non-repetitive fraction) and even increases the regional GC% values. This was not the case in the four mammalian genomes analysed. Since there is still no consensus about the origin of the AT/GC heterogeneity in vertebrates and the evolutionary mechanisms responsible, which may be varied [53], we assess our results in fish following the three main concepts discussed in [53]. First, the currently best supported view is that GC-biased gene conversion (gBGC) increases GC% at selectively neutral or weakly selected sites. Here, we can speculate that the small size of fish chromosomes might have resulted in a more effective gBGC through a higher rate of crossing over per Mbp [54,55] and led to GC-richness even in repeats. This should have, however, resulted in GC-richer genomes in fish than in mammals, which is not the case. Second, the high proportion of transposons in genomes results in a high rate of DNA methylation [56], and methylated cytosines are hypermutable and highly susceptible to spontaneous oxidative deamination [57,58], leading to a reduction in genomic GC% [59]. This could explain the observed homogeneous base composition of fish genomes. Moreover, the compact pufferfish genome, with low repeat and transposon density is GC-rich and heterogeneous. Finally, the role of selection in the GC evolution of the host genome [26,60] has largely been abandoned [53]. However, selection may play a role in the evolution of GC% of transposons and in their compositional interactions with host genomes. Here, it will be necessary to assess GC% first in functional and degraded transposons and in their different classes. The first results in this field show a higher GC% in the Class II transposons than in the Class I [52]. More importantly, there are indications that the base composition of human non-LTR retrotransposons is indeed evolving under selection and may be reflective of the long-term co-evolution between non-LTR retrotransposons and the host genome [61]. This study summarizes current knowledge on the base composition of transposons in mammals and its impact.

### 4.3. Towards Understanding the AT/GC Homogeneity of Fish Genomes

The inability to achieve G-banding in fish has been largely ascribed to their AT/GC homogeneity [29], and our detailed analyses of sequence data support this, albeit in only a small fraction of fish species (Table A1) covering 27 fish orders/groups (of the total 85; [62]).

There are no substantial differences among the here analysed teleosts indicating any so far hidden AT/GC heterogeneity, up to the role of genome size and repeats proportion in tetraodontiform fishes. On the other hand, a very special case is gars (Lepisosteiformes). These last survivors of an ancient lineage [62] were discovered to have a rather mammalian way of AT/GC heterogeneity [34]. In contrast, their most closely related, the last surviving species of Amiiformes, the bowfin (*Amia calva*, [62]), has the typical teleost-like AT/GC homogeneity [63]. These two fish groups still represent a puzzle that will persist at least until the genome assembly of bowfin will be available, which should be soon (Braasch, *pers. comm.*). At this stage, we can describe traits related to chromosome organization in the spotted gar–the only one gar species with a genome assembly available, luckily at the chromosome level [64]. Even more luckily, despite a high degree of incompleteness of the spotted gar’s genome assembly (945.878 Mb versus approx. C = 1.4 pg [40]), its GC-profile still clearly shows the mammalian type of AT/GC heterogeneity. The above-mentioned study on gars further compared CMA_3_-stained (i.e., GC-rich, red, AT-rich, green) chromosomes of selected vertebrate groups including the starry sturgeon (*Acipenser stellatus*). They show that the small-sized microchromosomes are red or reddish in this sturgeon, whereas macrochromosomes are homogenously green with reddish centromeres (Figure E in [34]). This corresponds to the results presented here (Figure 2 and Figure 3) in sterlet (*A. ruthenus*), where microchromosomes are GC-richer. This is an interesting result regarding the fact that numerous microchromosomes were presented with C-bands visualizing the constitutive heterochromatin in sturgeon hybrids [65]. On the other hand, these authors further present the results of their comparative genomic hybridization and genomic in situ hybridization showing the hybridization signals mostly on microchromosomes [65]. This might be alternatively interpreted that microchromosomes bear mostly coding regions that retain more sequence similarity among the compared species than the DNA on macrochromosomes that contain more repeats. Hence, clearly, this topic deserves further attention from both molecular cytogenetics and genomics to elucidate the potential differences between micro- and macrochromosomes. The importance of combining cytogenetics with genomics is evidenced by the fact that during sequencing, the first sturgeon microdissection of metaphase chromosomes assisted in proper genome assembly [27]. We address the quantitative traits/aspects of GC% in fish and across vertebrates in our other study published in this special issue [66].

Our results further show the GC-richness of small-size (micro)chromosomes also in three chondrichthyans, although the soft-masking did not work properly in the two of them (*P. pectinata* and *A. radiata*). There can be seen a great potential in comparisons with cytogenetic studies using CMA_3_-staining, e.g., [67] published an impressive AT/GC pattern in two *Scleropages* species (*S. jardinii* and *S. leichardti*), while the only species with an available genome (and processed here), *S. formosus*, appears to have the typical teleost AT/GC banding pattern [67]. This shows that the question of GC biology in fish and generally in vertebrates is still far from being solved satisfactorily.

## Data Availability

Publicly available datasets were analysed in this study. This data can be found at: https://github.com/bioinfohk/evangelist_plots.

## Appendix A

**Table A1:** Summarizing the fish species analysed in this study.

Species	Order	2n ^1^	Genome Size (pg) ^2^	GC%
*Acipenser ruthenus*	Acipenseriformes	120	1.8	39.8
*Amblyraja radiata*	Rajiformes	98	2.17	44.6
*Amphiprion percula*	Ovalentaria	48	0.9	39.5
*Astatotilapia calliptera*	Cichliformes	46	NA	41.1
*Astyanax mexicanus*	Characiformes	50	~1.5	38.4
*Betta splendens*	Anabantiformes	42	0.64	45.2
*Carassius auratus*	Cypriniformes	50	1.8	37.5
*Chiloscyllium plagiosum*	Orectolobiformes	102	~4.56	42
*Ciona intestinalis*	Tunicata	28	0.2	36
*Clupea harengus*	Clupeiformes	54	~0.9	44.2
*Cottoperca gobio*	Perciformes	48	NA	41
*Cynoglossus semilaevis*	Pleuronectiformes	44	0.62	41.3
*Cyprinus carpio*	Cypriniformes	100	1.8	37.1
*Danio rerio*	Cypriniformes	50	1.95	36.7
*Denticeps clupeoides*	Clupeiformes	40	NA	43.7
*Echeneis naucrates*	Carangiformes	48	0.7	41.4
*Erpetoichthys calabaricus*	Polypteriformes	36	4.7	40.1
*Esox lucius*	Esociformes	50	1.1	42.2
*Gadus morhua*	Gadiformes	46	0.65	46.3
*Gasterosteus aculeatus*	Gasterosteiformes	42	0.65	44.6
*Gouania willdenowi*	Gobiesociformes	48	NA	38.4
*Ictalurus punctatus*	Siluriformes	58	1	39.7
*Larimichthys crocea*	Perciformes	48	NA	41.4
*Lepisosteus oculatus*	Lepisosteiformes	58	1.4	40.1
*Maylandia zebra*	Cichliformes	46	NA	41.1
*Myripristis murdjan*	Beryciformes	48	~0.9	41.8
*Oncorhynchus mykiss*	Salmoniformes	58	2.7	43.4
*Oreochromis niloticus*	Cichliformes	46	1	39.9
*Oryzias javanicus*	Beloniformes	48	0.9	39
*Oryzias latipes*	Beloniformes	48	1	40.8
*Parambassis ranga*	Ovalentaria	48	NA	42.5
*Poecilia reticulata*	Cyprinodontiformes	46	0.88	40.3
*Pristis pectinata*	Pristiformes	92	2.8	42.6
*Salarias fasciatus*	Blenniformes	46	0.83	44.4
*Salmo salar*	Salmoniformes	60	3.15	43.9
*Scleropages formosus*	Osteoglossiformes	50	NA	44.1
*Scophthalmus maximus*	Pleuronectiformes	44	0.75	43.4
*Sparus aurata*	Perciformes	48	0.95	41.7
*Sphaeramia orbicularis*	Kurtiformes	48	NA	37.8
*Takifugu rubripes*	Tetraodontiformes	44	0.4	45.8
*Tetraodon nigroviridis*	Tetraodontiformes	42	0.43	46.6
*Xiphophorus maculatus*	Cypridontiformes	48	0.9	39.8

^1.^ Based on data in NCBI or Arai, 2011; ^2.^ Based on www.genomesize.com; NA, not available.
